# Multimodal Role of PACAP in Glioblastoma

**DOI:** 10.3390/brainsci11080994

**Published:** 2021-07-28

**Authors:** Agata Grazia D’Amico, Grazia Maugeri, Luca Vanella, Valeria Pittalà, Dora Reglodi, Velia D’Agata

**Affiliations:** 1 Department of Drug and Health Sciences, University of Catania, 95125 Catania, Italy; agata.damico@unict.it (A.G.D.); lvanella@unict.it (L.V.); valeria.pittala@unict.it (V.P.); 2 Section of Anatomy, Histology and Movement Sciences, Department of Biomedical and Biotechnological Sciences, University of Catania, 95100 Catania, Italy; graziamaugeri@unict.it; 3 MTA-PTE PACAP Research Group, Department of Anatomy, University of Pécs Medical School, 7624 Pécs, Hungary; dora.reglodi@aok.pte.hu

**Keywords:** glioblastoma multiforme, tumoral microenvironment, hypoxia, PACAP

## Abstract

Glioblastoma multiforme (GBM) is the deadliest form of brain tumors. To date, the GBM therapeutical approach consists of surgery, radiation-therapy and chemotherapy combined with molecules improving cancer responsiveness to treatments. In this review, we will present a brief overview of the GBM classification and pathogenesis, as well as the therapeutic approach currently used. Then, we will focus on the modulatory role exerted by pituitary adenylate cyclase-activating peptide, known as PACAP, on GBM malignancy. Specifically, we will describe PACAP ability to interfere with GBM cell proliferation, as well as the tumoral microenvironment. Considering its anti-oncogenic role in GBM, synthesis of PACAP agonist molecules may open new perspectives for combined therapy to existing gold standard treatment.

## 1. Introduction

Gliomas represent the most frequent malignant tumor affecting the central nervous system (CNS), including all glial cell-derived cancers. Based on World Health Organization classification, four types of gliomas can be classified: astrocytoma of grade I and grade I, representing astrocytic tumor, the grade III astrocytoma, consisting in anaplastic tumor, and grade IV astrocytoma or glioblastoma multiforme (GBM) [1]. The latter represents the deadliest brain cancer, with high cell heterogeneity and poor prognosis since it is characterized by therapeutic resistance and relapse after surgery. To counteract this issue, many studies have focused on identification of a new therapeutic approach consisting in co-administration of new molecules to the existing gold-standard treatment. In this way, the researchers have attempted to increase the therapy effectiveness, rendering tumoral tissue more vulnerable and counteracting its chemoresistance. Among new propositions, the inhibition of malignant cells infiltration into the surrounding parenchyma seems to be the more promising strategy. 

Pituitary adenylate cyclase-activating polypeptide (PACAP) is a pleiotropic peptide first isolated from ovine hypothalamus by Miyata and co-workers in 1989 [2]. This peptide is widely expressed in the CNS where it exerts different effects depending on the pathophysiological condition of tissue or organ [3,4,5,6,7]. Recent findings have demonstrated its involvement in various tumors, including GBM, where it exerts different effects depending on histopathological features of cancer [8,9,10,11,12,13]. In this review, we will first provide an overview of the GBM classification and existing therapy, highlighting the recent insight about the combined therapeutics approach reported in the literature. Finally, we will report existing findings related to PACAP involvement in GBM malignancy and the molecular mechanisms underlying its anti-oncogenic activity.

## 2. Glioblastoma Multiforme: Classification, Pathogenesis and Therapeutic Approaches

GBM is a lethal form of brain cancer affecting adults with poor prognosis, since the median survival of patients is between 14–17 months [14]. Based on different histologic features, the World Health Organization (WHO) classifies gliomas into astrocytoma of grades I, II, III or IV, the latter also being known as GBM [1,15]. By using genomic and transcriptomic analyses, GBM is also classified into four molecular subtypes [16,17]. Accordingly, a recent review has listed some criteria to subtype GBM, emphasizing their clinical relevance to develop a specific targeted therapy [18]. Based on expression of specific markers, it is possible to distinguish a Proneural, Neural, Mesenchymal or Classical GBM subtype [17]. In 2017, Wang et al. proposed a new classification including Classical, Proneural, and Mesenschymal GBM, since Neural subtype consist of non-tumor cells [19]. In accordance with this evidence, Teo et al. validated three robust GBM-subtypes: Proneural/Neural, Classical, and Mesenchymal by using a gene-classifier on six different platforms among various group population [20]. Moreover, large scale genomic studies extrapolated from The Cancer Genome Atlas (TCGA) have revealed various mutations on oncogene and/or onco-suppressor genes by subtyping GBM, including TP53, PTEN, Neurofibromin-1 and epidermal growth factor receptor (EGFR). In particular, the amplification of EGFR is detected in approximately 50% of primary tumors and occurs in the initial or recurrent stage. A specific EGFR mutation, known as EGFR variant III mutation, was detected in glioblastoma tissue in the initial phase as well as in relapse, although in the latter frequency was lower than in tumor tissue from the initial surgery [21,22]. Controversial findings concerning the correlation between tumor progression and EGFR amplification or EGFRvIII mutation are reported. Accordingly, some studies associated their alteration with an enhanced survival rate while others with bad prognosis [23,24,25]. Based on isocitrate dehydrogenase (IDH) enzyme mutations, it is possible to predict GBM outcome. In fact, characterization of IDH1 and IDH2 isoforms have assumed a prognostic value. In particular, patients carrying on mutations of these variants have a better response to standard treatment, as well as longer survival when compared to wild-type IDH1 patients [26,27,28]. An additional classification is based on identification of MGMT promoter methylation, involving 45% of GBM patients.

More recently, by following new recommendations proposed by cIMPACT-NOW research group, the upcoming WHO classification 2021 on CNS tumors discriminates between adult and pediatric GBM [29,30].

GBM is characterized by a heterogeneous mass, made up of infiltrating cells, stroma, blood vessels, secreted molecules and surrounding matrix. All these factors affect cancer development by promoting invasion and recurrence. In fact, cancer microenvironment contributes to tumor tissue transcriptional and genetic profile by comprising genes characterizing the different GBM molecular subtypes. A better understanding of tumor intrinsic signals, microenvironment and their interplay could help to understand the pathogenetic mechanism involved in malignancy and find a new combination for chemo- and immuno-therapy strategies to address a more specific individual therapy.

It has been largely demonstrated that microenvironment hypoxia represents a feature common to various solid tumors, including GBM, and it is linked to poor prognosis as well as therapy resistance [31,32,33]. In 1953, Gray and co-workers first demonstrated that hypoxic microenvironment determines tumor radio-resistance in different animal models [34]. Subsequently, other investigators have detailed this correlation [35,36]. More specifically, hypoxia contributes to the creation of an environmental cue directly responsible for cancer stem cells (CSCs) maintenance [37,38,39,40]. The latter escape radiotherapy or chemotherapy are directly responsible for cell genesis, aggressiveness, self-renewal and multipotency leading to cancer recurrence [41,42]. Furthermore, hypoxia reduces the expression of genes responsible for DNA repair, by inducing its mutations [43]; it also drives the transcription of hypoxia-inducible factors (HIFs), including HIF-1α and HIF-2α in GSCs. High HIF-1α levels were detected either in GBM GSCs or non-GSCs, whereas HIF-2α enhanced expression were reported exclusively in GSCs [44].

Hypoxia signaling cascade activates many downstream target genes responsible for enhancing cell invasiveness and uncontrolled neovascularization, including vascular endothelial growth factor (VEGF) [45]. Furthermore, it has been demonstrated that GSC population determines a perivascular induction of VEGF, which in turn leads to new vessel formation [46]. On the other hand, VEGF through an autocrine mechanism binds VEGFR2 receptor present on the GSCs surface, promoting the maintenance of the stem-like phenotype [35,47]. Another mechanism responsible for new vessel formation is the hypoxia-driven vasculogenesis. In this case, HIF-1α induces GSCs to secrete stromal cell–derived factor 1 (SDF-1), determining an increased attraction of heterogeneous population of bone marrow-derived endothelial progenitor cells, including endothelial progenitor cells (EPC), in tumoral periphery through mediation of CXC chemokine receptor type 4 (CXCR-4). The EPCs are involved in proliferation, as well as in trophic support for endothelial cells [48,49].

The effect of the tumoral hypoxic microenvironment is not limited to angiogenesis since it also regulates the transition of tumor epithelial cells towards the more malignant mesenchymal phenotype. The epithelial mesenchymal transition (EMT) is a key process involved in tumoral metastasis. Recent papers have demonstrated that the hypoxic microenvironment within tumor mass recruits circulating or residential myeloid cells (i.e., macrophages or microglia) into stroma, as well as triggers the activation of the EMT process [50,51,52].

Overall, the key role performed by the hypoxic microenvironment to drive progression of tumor towards malignancy by promoting different biological events is evident. Therefore, the use of drugs targeting tumor hypoxic pathways could improve radiation response in GBM patients [53].

Considering the structural heterogeneity of tumor mass, the actual approach consists of a multimodal treatment combining surgery, radiation and chemotherapy with additional molecules [15], also finalized to counteract recurrence.

Temozolomide (TMZ) is a molecule used in gold-standard treatment of GBM. This molecule is a DNA alkylating agent capable of inducing destruction of cancer cells since it prevents DNA replication. Usually, this drug is co-administered with radiotherapy for a further six cycles for maintenance [54,55]. Hepatic impairment and myelosuppression represent the most frequent side-effects [56].

Since VEGF overexpression is commonly found in GBM [57,58], targeting this factor is considered a promising therapeutic approach to counteract tumor progression [59,60]. Among the antiangiogenic proposed therapy, Bevacizumab (BV) has been approved by the US Food and Drug Administration (FDA) to treat GBM in adult [61]. It is a monoclonal antibody recognizing vascular endothelial growth factor (VEGF) and then capable of counteracting uncontrolled neovascularization. However, response to BV treatment seems to depend on the GBM molecular subtype [62]. A previous study demonstrated that BV was efficacious in patients with IDH1 wild type proneural glioblastoma [63], whereas it failed in some other treated patients [64]. Moreover, it has been reported that BV showed limited benefit on recurrent GBM, whereas it has no effects on the survival of patients with primary GBM [65,66]. This effect could be related to cells’ epithelial-mesenchymal transition (EMT), responsible of drug-resistance and tumor relapse [67,68]. Accordingly, Huang et al., 2017 [69], demonstrated in an in vitro study that BV is capable of increasing cell migration and EMT markers expression.

Recently, the effect of natural compounds, including cannabinoid [70] and an active natural bioflavonoid, Chrysin, have also been tested in GBM. The latter has been shown to exert an antiproliferative effect on glioblastoma cells [71]. Many other molecules have shown promising therapeutic effects, such as the inhibitor of epidermal growth factor receptor (EGFR), known as erlotinib. Unfortunately, many of these drugs do not cross the blood-brain barrier and, consequently, did not show any efficacy on patients’ survival during phase II of the clinical trial [72,73].

More recently, in an open-label phase 2 trial, known as REGOMA, researchers tested regorafenib, an oral multikinase inhibitor of angiogenic and oncogenic receptor tyrosine kinases, to treat recurrent glioblastoma [74,75,76,77]. It is noteworthy that Detti et al. reported an excellent response in a patient after three months of treatment with regorafenib [78].

## 3. PACAP and Its Related Receptors in Cancer

Pituitary adenylate cyclase-activating polypeptide (PACAP) is a peptide that belongs to the secretin/glucagon/growth hormone-releasing hormone/vasoactive intestinal peptide (VIP) family members, widely expressed in the CNS and in peripheral organs where it exerts different roles in a tissue-specific manner [79,80,81,82,83,84]. This peptide was isolated in 1989 from ovine hypothalamus [2]. It exists in two isoforms derived from the same precursor: PACAP-38, constituted of 38 amino acids, and PACAP-27 truncated in C-terminal domain, including 27 amino acids [85,86]. PACAP- 38 represents the predominant form in mammalian tissues. The various effects of PACAP are mediated by binding three different G-protein coupled receptors: PAC1 and VPAC receptors (VPAC1 and VPAC2). PAC1 shows higher affinity with PACAP rather than VIP, whereas both peptides show the same affinity to bind VPAC1 and VPAC2 receptors [87,88]. Different PAC1 receptor splice variants exist: Null, Hip, Hop1, Hop2, Hiphop1, Hiphop2, short and very short isoforms [89]. To date, several papers have summarized the distribution of PACAP and its receptors in different organs, including stomach, kidney, articular cartilage, human corneal endothelium, as well as in the CNS [90,91,92,93,94].

By binding to its receptors, PACAP can trigger different signaling pathways downstream adenylate-cyclase (AC) or phospholipase-C (PLC) activation, as well as calcium-regulated mechanisms [95]. In particular, PAC1 receptor is coupled either to G-protein alpha subunits Gs and Gq transmembrane receptors that mediate adenylyl cyclase/cAMP and phospholipase C (PLC)/DAG/IP3 signaling cascades, respectively [88,96]. More specifically, the PAC1 isoforms Null, Hop1, and Hop2 induce both pathways AC and PLC, whereas Hip isoform induces exclusively AC activation. The variants Hiphop1 and Hiphop2 represent an intermediate phenotype [97,98,99]. On the other hand, the VPAC subtypes are coupled predominantly to the Gαs transmembrane receptor that modulates cAMP signaling cascade [100,101].

Many studies have pointed out the role exerted by PACAP in some physiological conditions such as ageing, as well as in different pathologies, including neurodegenerative diseases, such as ischemia, traumatic brain injury, amyotrophic lateral sclerosis and retinopathy [102,103,104,105,106,107,108,109,110,111,112,113,114,115]. During the last decade, a lot of investigations have also reported the involvement of PACAP in different tumors, such as testicular, lung, breast, prostate, colon and pancreatic cancer, as well as neuroblastoma and glioblastoma [8,10,116,117,118]. However, its controversial role has been emphasized since it triggers different effects depending on the histopathological hallmarks of the tumor, the stage of disease as well as peptide concentration and time of treatment [119,120]. It is noteworthy that it has also been demonstrated that PACAP is capable of interfering with cancer progression even though it shows contrasting effects [87,121]. In fact, it promoted cell proliferation in some cases, whereas, in others, it reduced cell growth by inducing apoptotic cell death. Its endogenous expression, as well as the levels of its receptors seems to be dysregulated in the different neoplasms. More specifically, overexpression of PAC1 and VPAC1 receptors was detected in lung cancer, breast cancer, colon cancer, prostate adenocarcinoma, and pancreas tumor, whereas VPAC 2 receptor upregulation was detected in lung adenocarcinomas and neuroendocrine cancers [122,123,124]. However, reduced expression of both PACAP and its specific PAC1 receptor was described in pancreas adenocarcinoma samples [118] and reduced expression was measured with radioimmunoassay in lung, kidney and colon cancer samples in contrast to elevated levels in prostate cancer [125,126]. An altered staining pattern could be observed in other types of cancer, such as thyroid carcinoma and testicular cancer [117,127].

## 4. PACAP Involvement in GBM Malignancy

The presence of PACAP and related receptors was shown in human gliomas [128,129,130,131,132]. Recently, we have analyzed the peptide expression in human GBM samples by detecting lower endogenous PACAP concentration as compared to its receptors level [9].

The functional role of PACAP has been investigated by using several in vitro models of glioblastoma cells. In particular, an immunohistochemical study demonstrated that VPAC1 and VPAC2 are expressed in normal brain and glioma tissues by revealing a high cytoplasmatic expression related to grade of malignancy. The authors have also reported the differential nuclear localization of VPAC1, which increased with glioma grades, in contrast to weak nuclear staining of VPAC2 [133]. In 1996, Vertongen et al. [134] reported that PACAP significantly decreased proliferation of T98G human glioma cells; conversely, Sokolowska and Nowak (2008) [135] demonstrated that this peptide enhanced mouse C6 glioma cell proliferation after its exogenous administration. Considering these contrasting results, it has been hypothesized that the effect of the peptide depends on the origin species of studied cell lines. It has also been reported that PACAP treatment enhanced C6 glioma cell proliferation already at low concentration, ranging between 10^−15^ to 10^−13^ M [136]. Accordingly, our research group showed that PACAP increased C6 glioma cell viability already at 100 nM concentration after 48 h treatment [13]. By using two human glioblastoma cell lines isolated from different parts of a single tumor (known as M059K and M59j cells), it has also been proved that PACAP agonists reduced cancer cell migration, even though they did not affect their proliferation. Furthermore, these authors have shown that PACAP regulated cell invasion by acting through AKT signaling pathway [137,138]. Considering the highly invasive nature of GBM, these findings demonstrated that the potential anti-oncogenic property of this peptide is mediated through PAC1/VIPAC receptors activation. A recent study further characterized the molecular mechanism underling PACAP anti-invasive effect on GBM cells demonstrating that it acts by blocking PI3K/Akt and sonic hedgehog-GLI1 (Shh/GLI1) pathways [139]. The latter are the main signaling cascades responsible of GBM progression [140]. Furthermore, PI3K/Akt overactivation induced upregulation of MMP-2 and MMP-9, which in turn conferred to tumor cells the proteolytic capability to infiltrate normal tissue [141].

As mentioned above, the tumoral microenvironment plays a key role in cancer malignancy. It has been demonstrated that calorie restriction (CR) is efficacious in preventing cancer progression by increasing lifespan. In fact, it has been demonstrated that CR reduced cell proliferation by interfering with microenvironmental levels of several anabolic hormones, growth factors, inflammatory cytokines and oxidative stress agents [142]. We have investigated the effect of PACAP in C6 glioma cells cultured in serum free media, mimicking the microenvironmental tumoral condition under CR. Results have demonstrated that peptide treatment exacerbated CR-derived effects by leading to reduction of cell proliferation, as well as expression of nestin, a marker of cellular malignancy. This evidence allowed us to hypothesize that the effect of PACAP also depends on microenvironmental conditions [13].

**Figure 1:**
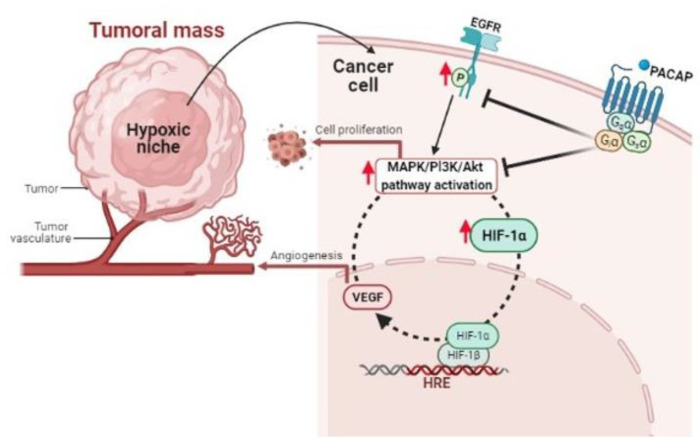
Schematic representation showing the PACAP modulatory effect in cellular microenvironment of hypoxic niches.

High cell proliferation produces in tumor mass hypoxic areas responsible for its malignant progression [143]. In these regions, we have demonstrated that PACAP acts by modulating the hypoxic pathway. Specifically, peptide treatment significantly reduced HIF-1α and HIF-2α levels in glioblastoma cells by inducing a drastic inhibition of PI3K/Akt and ERK1/2 signaling cascades, involved in uncontrolled cell proliferation. The hypoxic microenvironment also promotes the oncogenic program by inducing EGFR transactivation. This receptor is recognized as a prognostic marker of an advanced tumor stage since its anomalous expression is usually linked to reduced patient survival [144,145]. In our investigations, we found that PACAP also abrogated the aberrant EGFR transactivation occurring in GBM cells cultured in hypoxia, probably through inhibition of PI3K/Akt and MAPK/ERK pathway (Figure 1).

Recently, it has also been demonstrated that PACAP causes EGFR transactivation in non-small cell lung cancer in an oxygen-dependent manner that involves phospholipase C but not protein kinase A. Therefore, it could be hypothesized that the peptide’ effect in GBM may be mediated through a similar mechanism [146,147,148].

By using confocal laser scanning microscopy, we recently investigated the expression of PACAP and its high affinity related receptor, PAC1R, in hypoxic areas visualized on human GBM sections. PACAP co-localized only with HIF-1α, while PAC1R was present in hypoxic regions, as well as in tumoral stroma. Furthermore, PACAP or PAC1R co-localized with HIF1α in the cytoplasm or in the nucleus of tumor cells. This evidence confirmed a strict correlation between peptides and the hypoxic area in GBM. In this study, we have also demonstrated that PACAP interfered with uncontrolled neovascularization occurring in hypoxic niche. By using U87MG cells exposed to hypoxia, we observed that the peptide decreased VEGF intracellular expression, as well as its release in the growth medium. Culturing H5V endothelial cells in conditioned medium derived from GBM cells treated with PACAP under hypoxic condition, we observed a significant reduction in the number of tube-like structures, representing a model in vitro of micro-vessels formation. It is noteworthy that we have provided the first evidence demonstrating that the peptide interferes with the hypoxia-induced EMT process. As revealed by immunolocalization analyses, PACAP and PAC1R are expressed either in mesenchymal or in epithelial cells of GBM samples. Precisely, PACAP and its receptor co-localized in areas of human GBM expressing vimentin and MMP-2, two markers of mesenchymal phenotype, as well as in cells expressing ZO-1, a marker of epithelial phenotype. Moreover, in U87MG cells exposed to hypoxia, PACAP significantly reduced the mesenchymal phenotype markers, vimentin and matrix metalloproteinases MMP-2 and MMP-9, and, on the other hand, it increased ZO-1 expression [149]. This demonstrated that the exogenous administration of PACAP interfered with the EMT process by counteracting the epithelial cell differentiation towards mesenchymal phenotype. It has been recently reported that increased protein levels of MMP-2 and MMP-9 are responsible of extracellular matrix and basement membrane degradation allowing cancer cells to spread toward surrounding tissue [150]. In this regard, we have demonstrated that PACAP was able to counteract migration of U87MG cells exposed to hypoxia by interfering with the EMT event. In fact, it significantly reduced the number of Vimentin and CD44 immuno-positive migrating cells in the wounded area by using a model in vitro invasion assay. In accord, CD44 immunoreactivity is detected in about 55.55% of GBM, where its increased levels are related to shorter patients’ survival [151,152,153].

## 5. Conclusions

To date, data reported in the literature suggest that PACAP acts on GBM malignancy by interfering with cell proliferation as well as the tumoral microenvironment. In this study, we have highlighted its effect on modulation of HIFs pathway triggered in the hypoxic niches of tumor mass. The current therapeutical approach for GBM has many limits in terms of survival benefit. Therefore, the identification of new molecules capable of increasing glioma cell sensitivity to therapy might be desirable. Considering that PACAP does not cross the blood brain barrier, it could be helpful to synthetize new molecules targeting a peptide-driven signaling system to use in combination treatment with the existing therapeutic approach.
